# The Institutional Worker

**Published:** 1912-11-16

**Authors:** 


THE
### INSTITUTIONAL WORKER
**Being a Special Supplement to "The Hospital"**

### Editor's Notices.

**Contributions:**

Contributions should be written, or preferably typed, on one side of the paper only, and all articles sent in are accepted upon the distinct understanding that they are forwarded to The Hospital only.

The Editor cannot undertake to return MSS. not used, but when a stamped directed envelope is enclosed the MSS. may be returned if a special request is made.

Accepted articles and paragraphs of news will be paid for after publication at the scale rate.

**Address:**

To prevent delay all contributions and letters on editorial business must be addressed exclusively to the Editor, "The Hospital," 29 Southampton Street, Strand, London, W.C. It is important that this regulation shall be strictly observed.

**Correspondence:**

Correspondence on all subjects is invited. The name and address of correspondents must be given as a guarantee of good faith, but not necessarily for publication.

**Special Articles:**

Special articles are invited, and questions, inquiries, and paragraphs upon all matters relating to the work, administration, and management of general, special, mental, fever, cottage and convalescent hospitals, sanatoria, homes, institutions, societies and organisations for the treatment or care of the sick, injured and dependants of all classes. Every matter affecting the interests and welfare of the staff of all grades working in these institutions will receive special consideration.

**Administrative Medicine, Research and Items of News:**

Special terms will be paid for approved articles on subjects in this wide field by experts who have made some section of it their special study and interest. We wish to give prominence to every movement tending to advance accuracy of diagnosis, efficiency in treatment, and the development of modern methods to eradicate disease and promote the welfare of the suffering.

**A Bureau of Information:**

We invite inquiries and applications for information and help in providing for that numerous class of sufferers who require special care or house-room which they cannot obtain in their own homes or secure for themselves. **We cannot, however, prescribe, or recommend practitioners.**

**Books for Review:**

Publishers are particularly requested to send advance proofs of any new books of importance whenever possible, as the Editor has made arrangements to publish immediate reviews on a new plan.

**Photographs, Plans, Blocks, and Illustrations:**

It is requested that wherever possible MSS. may be accompanied by illustrations in any of the above forms. The name of the sender and of the article to which it belongs should be written on the back of each photograph, drawing, or block for purposes of identification.

**Local Papers:**

Newspapers containing reports or news paragraphs **should be marked** and addressed to the Sub-Editor.

**The Coupon System:**

A coupon will be found at the bottom of the third inside page of the cover each week. This coupon must be attached to every question or inquiry to which an answer is desired in The Hospital.

### Our Advertisement Pages.

Not the least of the many interesting and unique features of this week's issue of The Hospital is the large amount of useful information contained in the advertisement pages. Here will be found the results of commercial enterprise in its efforts to secure the most efficient appliances for the economical management of every department of institutional work. Consequently, the particulars contained in these pages cannot fail to prove of the utmost assistance to every keen and progressive institutional official. It is our purpose to make the pages of The Hospital as interesting and as comprehensive as possible, so that it is *the journal* of all that is essential and up to date in hospital and institutional affairs. In these circumstances, we would submit that our readers will find it to their interest to give some time and attention to our advertisement pages.

### Manager's Notices.

**All letters on business matters must be addressed to The Manager, "The Hospital," 28 and 29 Southampton Street, London, W.C.**

**Subscriptions**, which may commence at any time, are payable in advance. Cheques and Post-Office Orders should be crossed London County and Westminster Bank, Covent Garden Branch, and made payable to the Manager of The Hospital.

Rates of Subscription (payable in advance).

| | | |
|---|---|---|
| UNITED KINGDOM. | | |
| Three Months (including Postage) | ... ... ... ... | 2s. 0d. |
| Six Months do. | ... ... ... ... | 4s. 0d. |
| Twelve Months do. | ... ... ... ... | 6s. 6d. |
| FOREIGN AND COLONIAL. | | |
| Three Months (including Postage) | ... ... ... ... | 2s. 6d. |
| Six Months do. | ... ... ... ... | 5s. 0d. |
| Twelve Months do. | ... ... ... ... | 8s. 8d. |

**Advertisements:**

To ensure insertion, all advertisements for The Hospital must reach the Manager not later than Wednesday at noon in each week. For scale of charges see pages xxiii and 2.

**Special Rates** will be quoted for those institutions that agree to send all their public notices to The Hospital each year, so as to make it a file of such announcements for ready reference.

This form may be used for small prepaid advertisements, and when filled up should be posted to
*The Manager*, The Hospital,
28 and 29 Southampton Street,
Strand, W.C.

| | |
|---|---|
| Appointments Wanted, 21 words | ... 1s. 0d. |
| Appointments Vacant, 21 words | ... 2s. 6d. |

### The Scientific Press Publications

**LEDGERS, ACCOUNT BOOKS, STOCK BOOKS of all kinds for large and small Institutions.**

These are supplied already ruled and printed, or in accordance with the special requirements of institution managers.

***INSTITUTIONAL WORKS.***

### HOSPITALS AND ASYLUMS OF THE WORLD.

**By SIR HENRY BURDETT, K.C.B., K.C.V.O.**

In Four Volumes, with Separate Portfolio containing several hundred Plans.

**Vols. I. and II. (ASYLUMS) ... Price £6 15s.**

**Vols. III. and IV. (HOSPITALS) and the Portfolio of Plans ... Price £9**

**The Portfolio of Plans** (20 in. × 14 in., drawn to uniform scale), **Price £4 14 6**

**The Complete Work ... ... £12 12s.**

This magnificent work forms a Complete Survey of the Origin, History, Construction, Administration and Management of the Hospitals and Asylums of the World, with detailed information concerning the principal Institutions.

*Only a few copies remain unsold.*

**THE SCIENTIFIC PRESS, LTD., 28 and 29 Southampton Street, Strand, London, W.C.**

Send for complete Catalogue.

### Books for Institutional Workers.

"Midwife's Pocket Dictionary, with revised Rules of the C.M.B." ... 1s. 1d
"The Nurses' Pronouncing Dictionary" ... 2s. 0d
"A Handbook for Nurses." (J. K. Watson, M.D.) 5s. 4d
"Art of Feeding the Invalid." ... 1s. 6d.
"Surgical Ward Work and Nursing." ... 5s. 5d
"Massage Movements, including the Nauheim Exercises" ... 1s. 1d.
"Surgical Instruments and Appliances used in Operations" (Harold Burrows, M.B.London, B.S., F.R.C.S.) ... 1s. 8d.
"The Nurse's Materia Medica" (Herbert French, M.D.) ... 2s. 9d.

Case-papers, Charts, Professional Cards, and every kind of Institutional Literature.

Of all Booksellers or of The Scientific Press, Limited, 28 and 29 Southampton Street, Strand, London, W.C.

### HOSPITAL EXPENDITURE: THE COMMISSARIAT.

A USEFUL HANDBOOK FOR HOSPITAL MANAGERS AND MATRONS.

Crown 8vo, bound in cloth boards, **2s. 6d.** post free. Of all Booksellers, or of the Publishers—

THE SCIENTIFIC PRESS, LIMITED, 28/29 Southampton St., Strand, London, W.C.

